# A Comprehensive Machine-Learning Model Applied to Magnetic Resonance Imaging (MRI) to Predict Alzheimer’s Disease (AD) in Older Subjects

**DOI:** 10.3390/jcm9072146

**Published:** 2020-07-08

**Authors:** Gopi Battineni, Nalini Chintalapudi, Francesco Amenta, Enea Traini

**Affiliations:** Telemedicine and Telepharmacy Center, School of Medicinal and Health Products Sciences, University of Camerino, 62032 Camerino, Italy; nalini.chintalapudi@unicam.it (N.C.); francesco.amenta@unicam.it (F.A.); enea.traini@unicam.it (E.T.)

**Keywords:** MRI, machine learning, feature selection, ensemble methods, ROC

## Abstract

Increasing evidence suggests the utility of magnetic resonance imaging (MRI) as an important technique for the diagnosis of Alzheimer’s disease (AD) and for predicting the onset of this neurodegenerative disorder. In this study, we present a sophisticated machine learning (ML) model of great accuracy to diagnose the early stages of AD. A total of 373 MRI tests belonging to 150 subjects (age ≥ 60) were examined and analyzed in parallel with fourteen distinct features related to standard AD diagnosis. Four ML models, such as naive Bayes (NB), artificial neural networks (ANN), K-nearest neighbor (KNN), and support-vector machines (SVM), and the receiver operating characteristic (ROC) curve metric were used to validate the model performance. Each model evaluation was done in three independent experiments. In the first experiment, a manual feature selection was used for model training, and ANN generated the highest accuracy in terms of ROC (0.812). In the second experiment, automatic feature selection was conducted by wrapping methods, and the NB achieved the highest ROC of 0.942. The last experiment consisted of an ensemble or hybrid modeling developed to combine the four models. This approach resulted in an improved accuracy ROC of 0.991. We conclude that the involvement of ensemble modeling, coupled with selective features, can predict with better accuracy the development of AD at an early stage.

## 1. Introduction

Adult-onset dementia disorders are among the prevalent global medical issues in industrialized countries that have a high impact on individuals’ lifestyles. These disorders represent a great challenge for the community over their advancement from early diagnosis to end of life [1]. Statistical studies have estimated that every three seconds, a new dementia case is developing in the world. This means that approximately 50 million patients are suffering from this disease worldwide [1,2]. These numbers might double every twenty years and possibly reach 100 million patients by 2040.

Dementia is a syndrome that develops largely in older adults. It affects brain functionality, daily activities, and communication efficiency [1,3]. Alzheimer’s disease (AD) represents the prevalent form of adult-onset dementias. Some studies have highlighted that the early diagnosis of dementia is useful for starting treatments and for predicting outcomes of the disease but did not offer reliable methods for the early diagnosis of AD [4,5,6]. At the same time, some forms of mild cognitive impairment (MCI) do not evolve into overt dementia, whereas other forms of MCI represent a very mild form of AD [7]. In view of this, advanced computer techniques may represent a tool for the early diagnosis of AD and for predicting the evolution of prodromal forms of the disease or MCI into dementia.

Magnetic resonance imaging (MRI) techniques are becoming a relevant tool for prodromal AD and MCI evaluation [8]. A few studies based on the comparative analysis of cognitive testing and neuroimaging have hypothesized that AD neuroimaging may be enough to predict disease [8,9,10]. On the other hand, dementia forecasting with machine learning (ML) is becoming a more diffused approach in clinical practice [11]. In spite of the practical interest to quantify AD evolutions based on MRI data, only a few studies have calculated AD incidence rates based on MRI. 

Neuroimaging and primarily MRI provide essential information for AD dementia classification and prediction [12,13,14]. ML models, coupled with MRI information, can provide high diagnostic accuracy of age-related cognitive decline (ARCD) in dementia subjects [15]. It has been hypothesized that ML-supervised methods generate the knowledge of features necessary to correlate AD sample data [16]. It is also reported that logistic regression, coupled with cross-validation, can enhance the accuracy of AD prediction by speech amalgamation [17]. On the other hand, support vectors, along with feature reduction techniques, were able to classify dementia subjects with 70% accuracy [4]. 

The present study was designed to detect AD based on MRI findings along with the use of four ML models, such as naive Bayes, neural networks, k-nearest neighbor, and support vectors. Each model was validated separately by tenfold cross validation (CV). The receiver operating characteristic (ROC) curve value was used to evaluate the model accuracy. Three individual experiments were designed to test the model, and model performance was separately evaluated with given MRI characteristic information. The experiments that were done included

1. Models with manual selection of MRI features,
2. Models with automatic feature selection, and
3. A single model with ensemble learning or hybrid modeling.

The subsequent part of this paper is organized as follows. In Section 2, subject information of MRI features, feature selection techniques, and adopted models of AD prediction are analyzed. In Section 3, the experimental results of the four models are presented. In Section 4, each model is discussed and compared by accuracy and ROC parameters. Finally, Section 5 summarizes the main results of the present work.

## 2. Materials and Methods

### 2.1. Subjects

A longitudinal collection of 150 subjects and 373 MRI sessions was considered for this study. Each subject had undergone full screening of complete clinical assessment conducted at the Alzheimer’s Disease Research Center (ADRC) of Washington University. All subjects included, both men and women, were right-handed with a minimum age of 60 years and a maximum age of 96 years [18]. The subjects included 72 nondemented (ND) individuals and 64 demented (D) individuals (including 51 with mild to moderate AD). The remaining 14 subjects were identified as nondemented at the initial visit but resulted as demented when examined in subsequent visits. These subjects were defined as belonging to the converted (C) type.

Subjects undergoing age-related normal brain changes, such as leukoaraiosis, mild atrophy, and regular dementia cases of AD, were included in this study. All MRI sessions were done in one year. These sessions were followed by clinical tests made on 0–352 days (mean—111 days) after MRI. Twelve confirmed demented subjects were scanned with a delay ranging from 374 to 924 days (mean—653 days) and were included in this study as they had a clinical dementia rating (CDR) higher than zero in previous clinical assessments. Two nondemented subjects, with a scan delay range of 392 to 431 days, were also included because they did not display dementia symptoms in successive clinical evaluations. With this approach, each subject had at least two individual scan sessions with a mean delay of 719 days (range: 183–1707 days) between each visit. The demographic characteristics of the subjects are presented in Table 1. Diagnostic characteristics of subjects of different age groups on the initial clinical visit are detailed in Table 2.

### 2.2. MRI Acquisition Methods

Three or four separate T1-weighted MRI scans were acquired with a 1.5T Siemens Vision MRI scanner for each single subject. A high-resolution Magnetization Prepared Rapid Acquired Gradient Echo (MP-RAGE) was used to handle the classification of subject scans. For each subject, separate scan files were generated using Siemens proprietary IMA to 16-bit NiFTI1 format by employing the traditional conversion program. The MR images were corrected for interscan head rotation and wrapped spatially into atlas space. The transformation outcome placed the brains in a correlated coordinate system, with the bounding box as the actual atlas. With this procedure, every image was turned out as a unique, high contrast, averaged MP-RAGE image in an atlas-space. The insight explanation on image acquisition and postprocessing steps are detailed in [18]. 

The estimated total intracranial volume (eTIV) was defined manually across intracranial volume on an atlas. Normalized whole-brain volume (nWBV) was computed with the FAST program of the FSL software suite. Image segmentation was done to classify brain tissue as spinal fluid or white or gray matter. This segmentation process was iteratively assigned as voxels to tissue classes based on high probability estimates of hidden Markov random field models. In the end, nWBV was calculated as the proportion of accumulated voxels across the brain mask, and the normalized volume was expressed in a percentage of total gray and white matter voxels of eTIV [18]. The atrophy rates were estimated as the slope of the line that connects to nWBV. Details of the MRI acquisition characteristics are summarized in Table 3.

### 2.3. Feature Description

The dataset included 373 pieces of MRI information with 15 independent characteristics (attributes). The description of each feature is detailed in Table 4. The subject attribute “Group” specifies the dementia status (Demented/Nondemented) and is considered as an outcome of a binary classifier. In this study, scoring rules of Clinical Dementia Rating (CDR), Mini-Mental State Evaluation (MMSE), and Visit were used to determine the dementia status (Table 5). All subjects underwent similar procedures and received the same tests, including MMSE. 

### 2.4. Feature Selection

In this step, the machine performed an autonomous selection of input features that correlates to the subject group [19]. Selection techniques are largely used and standardized to reduce unnecessary features and to enhance model accuracy [20]. Moreover, this approach measures the relationship between independent variables and the target outcome. Feature selection can be conducted by three approaches, namely, filtering, regularization, and wrapping [20,21]. In this study, the wrapping technique was used because it amplifies model performance with limited features.

### 2.5. Feature Importance

**Figure 1:**
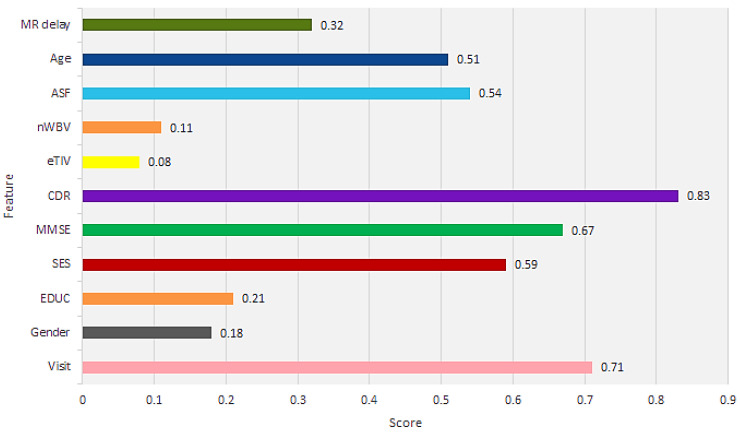
Individual feature scores.

This method results in a “feature score” assigned to independent characteristics and a defined score to each characteristic that is highly correlated with the subject “group”. The correlation between each characteristic-associated group variable is shown in Figure 1. The CDR rating was excluded during model development because it did not have the highest relevance, but it helps in subject groupings.

### 2.6. Feature Selection with Wrapping

**Figure 2:**
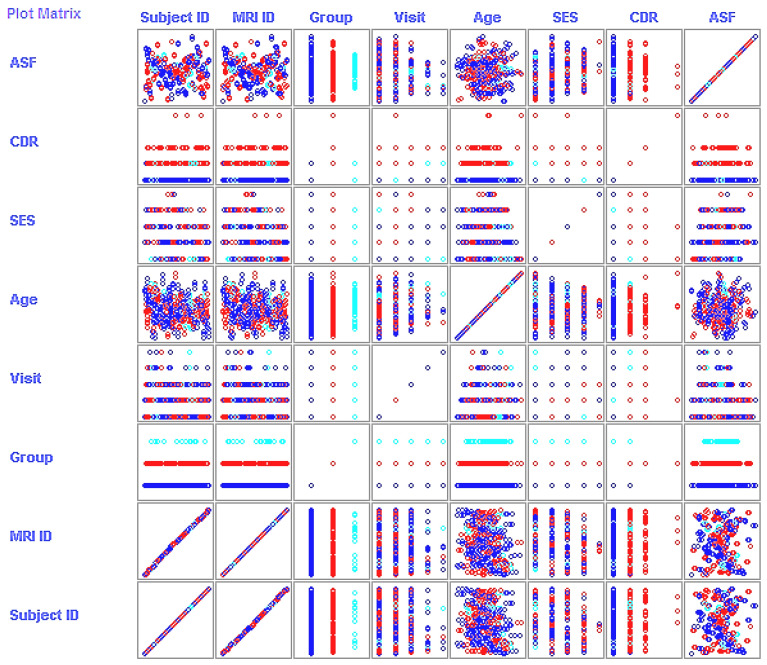
Scatter plot of selective features. Blue dots (ND), red dots (D), light blue dots (C).

In the wrapping method, feature search represents a big challenge in calculating model accuracy [22]. Feature selection can be made as either step backward or forward, and exhaustive. Feature search helps the identification of primary features in the enhancement of model performance. The MRI characteristics with a correlation of at least 0.5 can automatically help to develop a model. Figure 2 shows the scatter plot of feature results following the wrapping method.

### 2.7. Model Classifiers

The purpose of the present study is to develop a sophisticated ML model of dementia detection in aged subjects based on MRI findings. It is unanimously recognized that advanced age is the greatest risk factor for AD [23]. In this work, four popular ML models such as neural networks (NN) [24], k-nearest neighbor (KNN) [25], naive Bayes (NB) [26], and support vector machines (SVM) [27] were used. These models were selected because of the easy implementation and production of high accuracy during model development. A short description of each model is provided below. 

Neural networks are able to learn from independent features to predict target outcomes. They allow the design of an artificial neural network (ANN) to admit machines with the integration of new data [28]. ANN is largely associated with clustering (combining the unlabeled data of similar features) and classification (trained data grouping) procedures. One of the conventional and popular neural networks is the multilayer perception (MLP) type, which includes one or more neuron layers [29]. These neuron layers largely intervene to develop predictive models for forecasting clinical diagnoses [30]. 

KNN is a comprehensive model used to perform both regression and classification problems [25]. It is also called a “lazy” learner because instead of the model development approach, it calculates the nearest neighbors during prediction. When KNN initiates predictive analysis, it searches for nearest neighbors (i.e., K) in the trained dataset. The neighboring distance is then calculated with the Euclidean function, which defines the similarity between two points [31].

NB is a probabilistic model that predicts output based on Bayes’ principle. It calculates the outcome value of individual groups, which is not associated with other variables [26]. Due to its simplicity during target prediction, it has become popular in classification and multiclass predictions [32].

SVM is another algorithm developed for subject classification. In SVM plotting, dataset features are described in n-dimensional space (here, “n” is feature count), and classification is done to decide the optimal hyperplane [27]. In more detail, SVM produces an optimal hyperplane with the trained label data that classifies new feature examples. This hyperplane is a line of binary classification and tuning parameters, such as “kernel”, “gamma”, and “C”, that can help to improve SVM model performance [33,34].

### 2.8. Performance Measures

After model development, it is important to evaluate individual model performance. This is calculated through the prediction of the trained model of a test dataset. Different parameters like accuracy (A_cc_), sensitivity (S_e_), specificity (S_p_), and receiver operating characteristic (ROC) curve define model performance. To calculate each parameter, the confusion matrix (CM) was used to identify misclassifications in tabular form (Table 6). A subject is true-positive when it is diagnosed as demented (X = D), and a subject is true-negative when it diagnosed as “nondemented” (Y = ND).

The performance measures evaluated by CM are given below:

- Accuracy: Percentage of total true predicted outcomes from total outcomes, i.e., Accuracy (%) = $\left(\frac{\mathrm{TP}+\mathrm{TN}}{\mathrm{TP}+\mathrm{TN}+\mathrm{FP}+\mathrm{FN}}*100\right)$.
- Sensitivity: It measures the proportion of true-positives, i.e., Sensitivity (%) = $\left(\frac{\mathrm{TP}}{\mathrm{TP}+\mathrm{FN}}*100\right)$.
- Specificity: It measures the proportion of true-negatives, i.e., Specificity (%) = $\left(\frac{\mathrm{TN}}{\mathrm{TP}+\mathrm{FN}}*100\right)$.
- ROC: ROC is a performance visualization tool of binary classifiers with the false-positive rate (FPR) on the *X*-axis and the true-positive rate (TPR) on the *Y*-axis. In this study, we mainly highlight the ROC value to determine model performance because it is frequently used in medical diagnosis.

### 2.9. Model Validation and Framework

**Figure 3:**
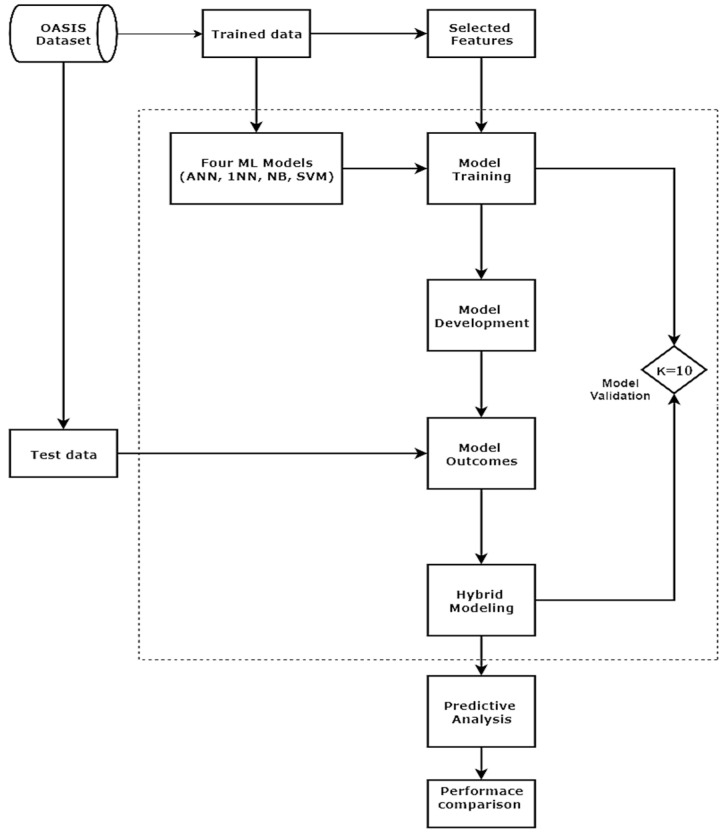
Model framework for evaluating predictive classifications.

Model validation can be done by either holdout (spilt) or cross-validation (CV) techniques. During this study, we adopted the CV technique because of its popularity in target prediction, with low bias. Simultaneously, it also applies a resampling method with limited features during model validation [35]. In CV, the dataset is distributed into N-folds of equal size. The first fold is used for validation, and the remaining k-1 folds are kept for training. The model framework used during simulation is represented in Figure 3. 

### 2.10. Experiments Design

A large number of MRIs for a low number of subjects could generate bias in dementia detection. Therefore, we considered final MRI scans that define the status of each subject. Three experiments were conducted, including manual and automatic feature selection techniques.

In the first experiment, model training was done using the original dataset with manual feature selection. In ANN, the number of layers (N) is used as a search parameter during model evaluation. In KNN, k is tuned to one (i.e., 1NN). In SVM, the linear kernel coupled to regularization parameter “C” and a standard deviation of radial basis function “r” are implemented in model tuning. Finally, model validation was done with a 10-fold CV to avoid data fitting issues [36]. The model performance was, therefore, assessed by the above parameters.

In the second experiment, limited features that occurred as the result of wrapping were considered for conducting model training. For NB and KNN, an exhaustive search was used to calculate model accuracy with potential feature alliance in order to select the best of them [37]. In SVM, genetic algorithms (GAs) were used for the feature search. GAs are frequently applied in bioinformatics to generated models with high accuracy [38]. For ANN, the feature search was excluded, and the search consisted of the identification of the hidden neuron layers. Model tuning was adjusted by maintaining batch size as 100 in NB, (C, gamma) as (1.0, 1.0 × 10^−12^) in SVM, and k = 1 in KNN. MRI characteristics that were highly correlated (≥0.5) with subject groups were selected (see Figure 2).

In the third experiment, the four models were combined to develop an ensemble or hybrid model. By doing this, there is the advantage of getting a high prediction accuracy of the adopted dataset. Moreover, combining several models can enable noise reduction (bagging), low bias (boosting), and better predictions (voting). We used a voting technique in this experiment because of the capability to create standalone models from trained data [39].

## 3. Results

### 3.1. Experiment 1: Handling of the Feature Set Prior to Autonomous Feature Selection

Table 7 summarizes the performance outcomes of the four models in manual feature selection. The CDR rating was excluded as it represents a dementia factor that can affect model accuracy. From the performance comparison matrix, it can be seen that the 1NN model offers better performance compared to the other tested models in terms of accuracy, sensitivity, and specificity. As already mentioned, the ROC curve plays a relevant role in diagnostic assessments to differentiate the true state subjects and to find optimal cutoff values. Moreover, a higher ROC offers better dementia prediction in given subjects [40]. In view of this, the ANN model correctly discriminates against the true demented subjects, with a ROC of 0.812. The ROC of NB, 1NN, and SVM models produced ROCs of 0.753, 0.787, and 0.796, respectively. 

### 3.2. Experiment 2: Automatic Feature Selection with Wrapping

Table 8 shows the model performance outcomes obtained with automatic feature selection. With this approach, progress in terms of accuracy and ROC compared to manual feature selection was noticeable. SVM resulted in high accuracy (96.12%), and 1NN, NB, and ANN produced an accuracy of 95.92%, 93.44%, and 83.56%, respectively. With regard to ROC, NB was a better diagnosis predictor, with 0.942, followed by 1NN, SVM, and ANN, with 0.916, 0.834, and 0.817, respectively. 

The results of the present experiment, in which performance results were better than those obtained in the previous one, stimulated the identification of other approaches for maximizing prediction accuracy. We, therefore, extended our work to explore the outcomes of joint modeling with limited features. 

### 3.3. Experiment 3: AD Predictions with Hybrid Modeling

**Figure 4:**
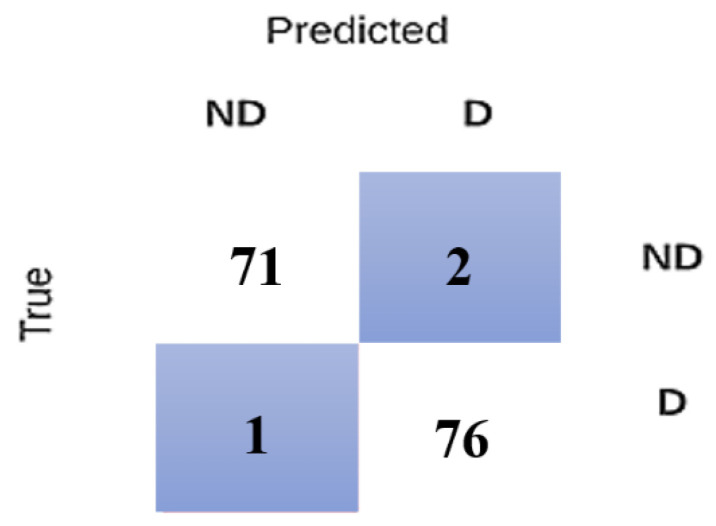
Confusion matrix outcome of the hybrid model (D: Demented; ND: Non demented).

To check if a model correctly predicted the target variable (occurrence of dementia), a confusion matrix was used. In this analysis, vertical labeling presents actual subjects, and horizontal labeling presents predicted subjects. As shown in Figure 4, 76 subjects were correctly predicted as AD among 78 subjects, and 71 subjects were correctly predicted as non-AD among 72. Collectively, 147 subjects were properly predicted out of 150 subjects. This results in 98% accuracy. For reaching these conclusions, a hybrid-modeling technique, combining the four adopted models, was introduced.

**Figure 5:**
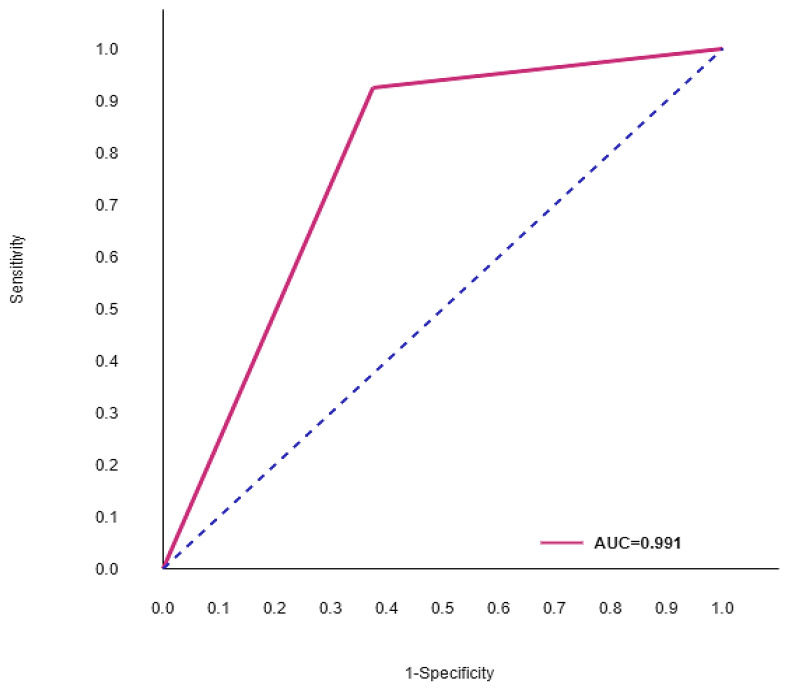
Receiver operating characteristic (ROC) curve of the hybrid model.

The performance of the individual subject group is presented in Table 9. Nondemented and demented subjects were correctly diagnosed with 98.6% and 97.4% accuracy, respectively. The weighted average ROC curve of both subjects nearly touches one. Hence, maximum AD subject predictions have been made without bias because of hybrid modeling. The sensitivity and specificity rates produced were 98.05% and 98%, respectively. The ROC curve of the hybrid model is shown in Figure 5. Based on the evaluation of performance differences in the above three experiments, the intervention of hybrid modeling with limited features resulted in being good practice in AD-related studies.

## 4. Discussion

ML models are highly acknowledged in real-time clinical practice and also in diagnosis and AD treatment selection [41]. Several MRI works have been integrated into ML models to make AD predictions [12,17,42], but there has been no comprehensive model to amplify model accuracy. In view of this, we introduced a hybrid model to enhance the precise detection of AD based on the analysis of MRIs.

**Figure 6:**
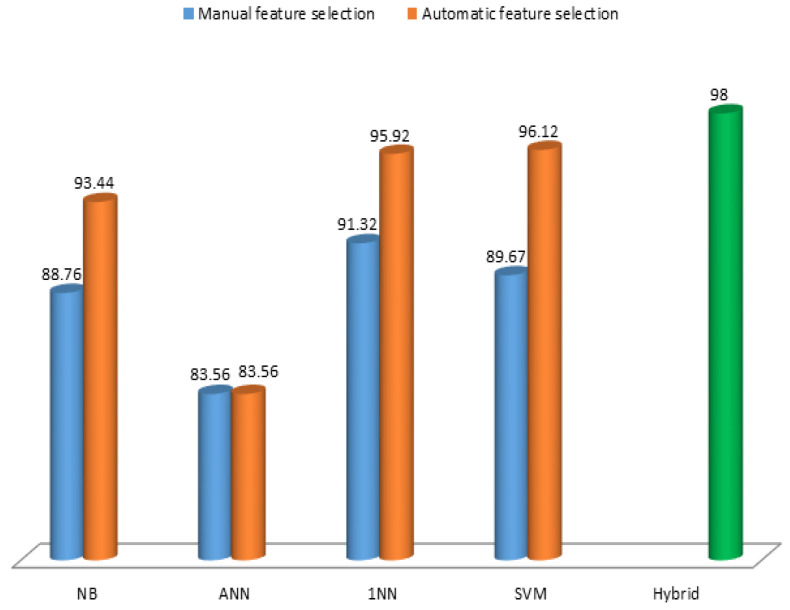
Prediction accuracy (in %) comparisons of three experiments.

In this paper, the significance of joint ML modeling for AD-onset prediction in elderly people has been demonstrated. Three different experiments were conducted, including manual and automatic feature selection techniques. Fourteen independent MRI features were used to identify the AD group using standard diagnostic approaches. Four supervised predictive models (NB, ANN, KNN, and SVM) were used, and the obtained results indicate the prediction accuracy of each model, constantly increasing between experiments. Figure 6 compares the prediction accuracy of the three experiments. 1NN generated 91.32% accuracy by manual feature selection; SVM had a high 96.12% accuracy by automatic feature selection, whereas joint or hybrid modeling enabled 98% accuracy in predicting AD in older adults. The outcomes suggest that joint modeling, with limited features, is a best practice to assess AD-onset by subject prediction. 

In the first experiment, all the designed classifiers revealed enough performance values in terms of true-positive rates (sensitivity). ANN and 1NN produced the highest sensitivity (89.92%), followed by SVM (89.24%) and NB (82.43%). As mentioned, ROC curve values between 0.5 and 0.7 indicate low prediction accuracy, between 0.7 and 0.9 indicate moderate prediction accuracy, and between 0.9 and 1 indicate high prediction accuracy [43]. From Table 7, it is obvious that the four adopted models produce moderate prediction accuracy when checking with manual feature selection.

**Figure 7:**
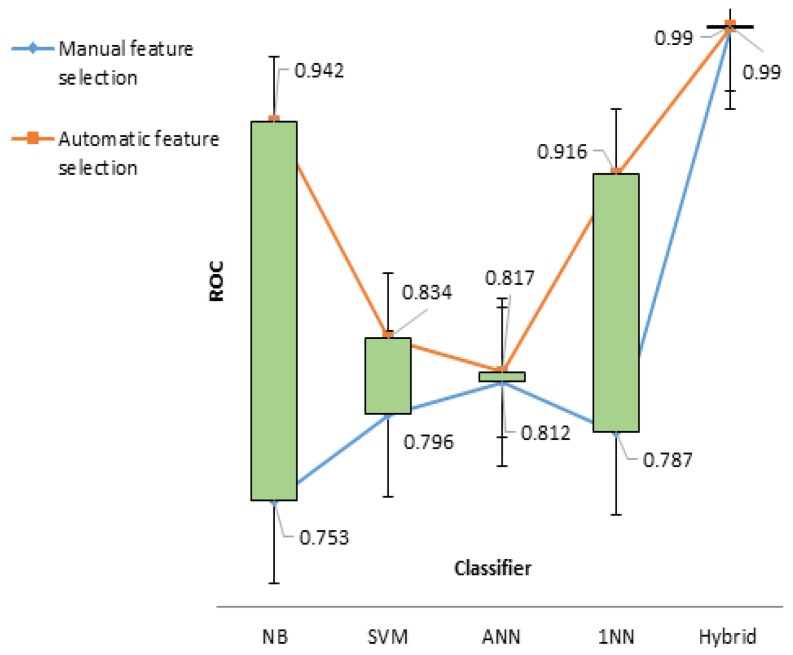
ROC comparison of hybrid modeling with other experiments.

To amplify model performances, the second experiment was conducted with selective features after wrapping. This resulted in NB of 98.21% sensitivity, followed in descending order by SVM (94.94%), ANN (94.92%), and 1NN (89.92%). Both NB and 1NN predict subject class in a comparatively better manner, with ROC of 0.942 and 0.916, respectively. However, we argued that there could be other possibilities for enhancing prediction accuracy to values higher than those identified in the above two experiments. To support this claim, a hybrid model was developed by combining the four investigated models. A simulation of four recruited models was then performed, and thanks to this approach, the sensitivity of the model attained the highest predicted value of 97.4%, and its ROC was nearly equal to one (Figure 7).

The developed model produced better accuracy than other conventional models, but the present study has some limitations. First, the limited number of subjects investigated could hamper the final dementia subject prediction to the overall AD subjects; second, the outcome of the integration of three experiments may have influenced the results. The use of external MRI information does not guarantee data quality and can affect the significance of the study as a whole.

Brain studies corroborated with artificial intelligence analysis may offer relatively faster investigation methods to modern neurological research. However, it would be preferable to avoid data limitations and, therefore, to enlarge as much as possible the size of the sample investigated in future studies. At the same time, it is also recommended to apply hybrid modeling to younger subjects or subjects with mild AD and to anticipate prediction accuracy with other biological tests like cerebrospinal fluid (CSF) or blood markers.

## 5. Conclusions

Adult-onset dementia disorders are serious brain pathologies caused by the loss of neuron functions and to progressive atrophy. AD is the most common of these pathologies. It affects primarily elderly people and has a tremendous impact on the lives of people suffering from it. In view of the long time passing between brain lesions bringing about dementia and the onset of clinical symptomatology, early identification of the preclinical and prodromal forms of the disease represents a challenge for medicine. This will reduce medical costs and could contribute to undertaking therapeutic approaches for delaying the conversion of the disease into overt dementia.

Unfortunately, the identification of AD at very early stages is extremely difficult, and there are no tools for its simple detection. We have developed different ML models to predict dementia in the elderly based on MRI findings. The hybrid model with selective features was found to enhance the accuracy of dementia prediction. Experiments with manual feature selection prior to automatic feature selection with 1NN produced 91.32% of accuracy, and the experiment of automatic feature selection generated 96.12% accuracy by SVM. This value significantly increased using multi modeling and produced 98% accuracy. The predictive models developed in this study forecast early AD diagnosis and the associated risk of developing dementia. Although it is difficult to develop longitudinal projection models in older adults as compared to the younger population, future research in the field should consider addressing both genetic and nongenetic features of multifactorial hazards.

## Figures and Tables

**Table 1:** Demographic characteristics of the subjects investigated.

**Subjects**	78 D	72 ND
**Male**	40 D	22 ND
**Female**	38 D	50 ND
**Age range (years)**	60–96	
**Median**	77.0	
**Mean ± SD**	77.01 ± 7.3	

D: demented; ND: nondemented; SD: standard deviation.

**Table 2:** Age and characteristics of the individuals investigated on the first clinical visit [18].

		Non-Demented					Demented				
Age Group	N	n	Mean	Male	Female	Convert	n	Mean	Male	Female	CDR 0.5/1
60s	34	23	65.71	6	17	3	11	65.67	8	3	8/3
70s	71	35	74.91	11	24	4	36	73.97	20	16	29/7
80s	41	26	84.30	9	17	7	15	82.33	7	8	13/2
90s	4	2	92.50	0	2	0	2	93.00	1	1	1/1
Total	150	86	75.82	26	59	14	64	74.95	36	29	52/13

CDR: clinical dementia rating.

**Table 3:** Magnetic resonance imaging (MRI) acquisition details [17].

MR Characteristics	Values
Sequence	MP-Rage
TR (repetition time)	9.7 msec
TE (echo time)	4.0 msec
Flip angle	10°
TI	20 msec
TD	200 msec
Orientation	Sagittal
Thickness	1.25 mm
Gap	0 mm
Slice number	128
Resolution	256 × 256 (1 × 1 mm)

MP-RAGE: Magnetization Prepared Rapid Acquired Gradient Echo; TI: Inversion time; TD: Dead time.

**Table 4:** Dataset feature description.

Features	Description
Subject ID	Subject identification number
MRI ID	Image identification number of an individual subject
Visit	Number of subject visits
Gender	Male/Female
Hand	Right/Left handed
EDUC	Subject education level (in years)
SES	Socioeconomic status
MMSE	Mini-mental state examination score
CDR	Clinical dementia rating score
eTIV	Estimated total intracranial volume result
nWBV	Normalized whole brain volume result
ASF	Atlas scaling factor
Age	Subject age while scanning
Group	Demented/Nondemented/Converted
MR delay	Magnetic resonance (MR) delay is the delay time that is prior to the image procurement

**Table 5:** Scoring rules.

Features	Range	Condition
CDR	0–3	None—0, Very mild—0.5, Mild—1, Moderate—2, Extreme—3
MMSE	1–30	Extreme impairment (<10)
		Moderate dementia (10–19)
		Early-stage Alzheimer’s aliment (19–24)
		Normal (>25)
Visit	0 or 1	Low status—0High status—1

**Table 6:** Simple confusion matrix (CM).

Prediction	X	Y
**X = D**	TP	FN
**Y = ND**	FP	TN

D: demented; ND: nondemented; TP: true-positive; TN: true-negative; FP: false-positive; FN: false-negative.

**Table 7:** Performance comparison matrix (4 × 4) of four classifiers.

Model	Accuracy (%)	Sensitivity (%)	Specificity (%)	ROC
NB	88.76	82.43	85.72	0.753
ANN	83.56	89.92	88.84	0.812
1NN	91.32	89.92	89.56	0.787
SVM	89.67	89.24	89.45	0.796

NB: naive Bayes; ANN: artificial neural networks; 1NN: 1-nearest neighbor; SVM: support vector machines; ROC: Receiver operating charactersitcs.

**Table 8:** Model performance evaluation after feature selection (with selective features).

Model	Accuracy (%)	Sensitivity (%)	Specificity (%)	ROC
NB	93.44	98.21	97.32	0.942
ANN	83.56	89.92	88.84	0.817
1NN	95.92	94.92	97.36	0.916
SVM	96.12	94.94	98.23	0.834

**Table 9:** Performance statistics of hybrid modeling.

Accuracy (%)	Sensitivity (%)	Specificity (%)	ROC	Class
98.6	98.7	98.6	0.992	ND
97.4	97.4	97.4	0.989	D
98.0	98.05	98.0	0.991	Weighted average
